# Quantitative Characterization of Smooth Pursuit Eye Movements in School-Age Children Using a Child-Friendly Setup

**DOI:** 10.1167/tvst.8.5.8

**Published:** 2019-09-11

**Authors:** Valldeflors Vinuela-Navarro, Jonathan T. Erichsen, Cathy Williams, J. Margaret Woodhouse

**Affiliations:** 1 Aston Optometry School, Aston University, Birmingham, UK; 2 School of Optometry and Vision Sciences, Cardiff University, Cardiff, UK; 3 Population Health Sciences, Bristol Medical School, Bristol University, Bristol, UK

**Keywords:** smooth pursuit, gain, development, child-friendly

## Abstract

**Purpose:**

It could be argued that current studies investigating smooth pursuit development in children do not provide an optimal measure of smooth pursuit characteristics, given that a significant number have failed to adjust their setup and procedures to the child population. This study aimed to characterize smooth pursuit in children using child-friendly stimuli and procedures.

**Methods:**

Eye movements were recorded in 169 children (4–11 years) and 10 adults, while a customized, animated stimulus was presented moving horizontally and vertically at 6°/s and 12°/s. Eye movement recordings from 43 children with delayed reading, two with nystagmus, two with strabismus, and two with unsuccessful calibration were excluded from the analysis. Velocity gain, proportion of smooth pursuit, and the number and amplitude of saccades during smooth pursuit were calculated for the remaining participants. Median and quartiles were calculated for each age group and pursuit condition. ANOVA was used to investigate the effect of age on smooth pursuit parameters.

**Results:**

Differences across ages were found in velocity gain (6°/s *P* < 0.01; 12°/s *P* < 0.05), as well as the number (12°/s *P* < 0.05) and amplitude of saccades (12°/s *P* < 0.05), for horizontal smooth pursuit. Post hoc tests showed that these parameters were different between children aged 7 or younger and adults. No significant differences were found across ages in any smooth pursuit parameter for the vertical direction (*P* > 0.05).

**Conclusions:**

Using child-friendly methods, children over the age of 7 to 8 years demonstrated adultlike smooth pursuit.

**Translational Relevance:**

Child-friendly procedures are critical for appropriately characterizing smooth pursuit eye movements in children.

## Introduction

Evidence from previous published studies suggests that saccades (eye movements responsible for fast shifts of gaze from one object of interest to another) achieve adult values early in life.^1^–^4^ For instance, saccadic duration and peak velocity are not significantly different between adults and children aged 5 or older.^2^–^4^ In contrast, smooth pursuit (eye movements responsible for the smooth tracking of moving objects) has been suggested to continue its development throughout infancy and childhood.^5^–^8^

It is generally accepted that, in infants, pursuit is mainly achieved by sequences of saccades, but these decrease very early in life, at which point pursuit starts to be mainly dominated by smooth pursuit eye movements.^9^,^10^ Although there is significant development in the first months of life, smooth pursuit performance seems to be still well below adult levels at the age of 1 year.^10^,^11^ One of the largest studies to date evaluating the development of smooth pursuit in children showed that the proportion of time devoted to smooth pursuit (i.e., without saccades) and velocity gain both significantly increase between 1 and 6 years of age.^11^ Unfortunately, this study did not include an adult group, and therefore it cannot be determined when smooth pursuit eye movements reach adultlike levels. Results from a different study evaluating this type of eye movements in older children (aged 7–12 years), which also included an adult sample (aged 30–38 years), suggested an immaturity of the smooth pursuit system in school-age children.^12^ Although the results indicated that older children have velocity and position gains very similar to those found in adults, their eyes still did not match the position and velocity of the target as accurately as adults did.^12^ The study concluded that, even at the age of 12 years, children's pursuit eye movements are still immature.^12^ In contrast, other authors have reported negligible differences in gains between adults and children aged 7 to 11 years.^3^,^5^,^11^ Hence, while smooth pursuit eye movements are unarguably not mature and adultlike in infants and young children aged 4 to 7, the debate continues about their development and maturation levels after that age.

There are a number of possible arguments that can explain the conflicting results found in the literature. In general, eye movement parameters have been reported to be more variable within a group of children than within a group of adults (e.g., see Refs. 5, 12, 13). This variability might arise from different maturation levels within children of the same age and/or external factors, including attention and fatigue, that can strongly interfere with smooth pursuit performance.^12^ In addition, most studies investigating the development of pursuit in childhood have used traditional stimuli to elicit these eye movements (e.g., dots and light spots),^4^,^7^,^12^,^14^,^15^ but recent evidence has shown that children respond better to more interesting targets resulting in improved performance, and therefore improved oculomotor responses.^3^ In addition, our group has demonstrated that smooth pursuit performance can be improved even in a young adult population using a dynamic stimulus (i.e., an animated stimulus).^16^ Therefore, it can be argued that studies investigating smooth pursuit performance in children using traditional stimuli do not provide an optimal measure of eye movement characteristics in this population. Hence, the purpose of the study was to quantitatively characterize smooth pursuit performance in school-age children (4–11 years) using a child-friendly, animated stimulus that has been shown to improve eye movement performance in an adult population.^16^

## Methods

### Participants

One hundred sixty-nine school-age children (75 females and 94 males) ranging in age from 4 to 11 years were recruited from two schools in South Wales (UK). At the end of the study, 43 (14 females and 29 males) of the recruited children were identified by their teachers to have delayed reading skills and/or poor academic performance, and their data were discarded. The remaining children (*n* = 126; 61 females and 65 males) were classified into groups according to their age at the time of the study. Each age group consisted of a minimum of 12 children (mean number of children per group: 15 [SD ± 5.7]), except for the 11-year-olds. This group consisted of only three children aged 11 as further recruitment was not possible due to the busy schedule and learning programme in the last school year. Ten young adult subjects (5 females and 5 males) with a mean age of 22.60 (SD ± 2.31), who were undergraduate students at the School of Optometry and Vision Sciences, Cardiff University, were also recruited to obtain adult data for comparison.

All study participants were screened prior to the eye movement recording to confirm visual acuity of logMAR ≤0.1 with spectacle correction (if any), no strabismus, no manifest refractive errors of more than 8.00 diopters (D), and no accommodative problems (i.e., accommodative lag ≤1 D at 25 cm). The aim of this screening was to exclude participants with any obvious optometric deficits that could impact on the participant's ability to see the eye movement targets clearly. None of the participants wore contact lenses during the eye movement recording.

The protocol for the study as well as the study information sheets, consent forms, and opting out forms were approved by the Cardiff University School of Optometry and Vision Sciences Ethics and Audit Committee, and were designed in accordance with the Declaration of Helsinki. Written informed consent or opting out forms (as preferred by each school) were obtained from the child participant's parents or guardians. Written informed consent forms were obtained from the adult participants.

### Eye Movement Recording

#### Procedures

Eye movement recordings were obtained under binocular conditions using the Tobii TX300 (Tobii Technology, Stockholm, Sweden) eye tracker.^17^ Participants were seated 65 cm away from the computer monitor and the eye tracker with their eyes in primary position, and facing the center of the computer monitor. The Tobii TX300 eye tracker gaze accuracy given by the manufacturer is ±0.5° for monocular and ±0.4° for binocular conditions.^17^ The child-friendly head stabilizer previously described by the authors^16^ was intended to be used by all the study participants (including the adult group) to encourage them to maintain their head at a constant distance from the eye tracker and the screen throughout the test. However, some children aged 10 to 11 preferred not to use the head stabilizer. In this situation, the researchers explained to the child the importance of keeping the head still at the same distance from the screen throughout the test.

Tobii Studio (Tobii Technology, Stockolm, Sweden) was used to calibrate and record the eye movements of each child participant. Prior to the eye movement recording, the eye tracker was calibrated for each participant using the standard Tobii five-point calibration. During the calibration procedure, Tobii Studio presented a target (red dot) that randomly moved to five points on the screen, including the geometric center and the four corners of the screen. All stimuli presented later were contained within the calibrated area, which corresponded to the entire widescreen display area.

#### Visual stimuli and smooth pursuit tasks

The animated stimulus developed previously by our group^16^ was used to record horizontal and vertical smooth pursuit in all participants. In brief, the animated stimulus consisted of a 2° animal cartoon image that moved horizontally or vertically, while continuously changing shape and color as it morphed into different animals.^16^

An initial start button was displayed in the center of the screen before the test began. When the participant was ready, the researcher clicked the start button and the smooth pursuit test started with the animated stimulus appearing for 1 second at 10° to the left of the participant's straight-ahead position. Then, the stimulus moved horizontally (left to right) following a constant velocity motion of 6°/s. The stimulus stopped when it was at 10° to the right of the participant's straight-ahead position. Two-second fixation periods were presented between each ramp (left to right or right to left) before the stimulus moved again to the left or to the right. Four smooth pursuit ramps were presented, so that the stimulus moved left to right and right to left twice. Then, the same animated stimulus was presented following the same horizontal motion, but at a velocity of 12°/s. Finally, the animated stimulus was presented moving vertically following the same motion paradigm and velocities (6°/s and 12°/s).

### Data Analysis

Once the data from all the participants were collected, the eye position traces were extracted from Tobii Studio and analyzed offline using custom software written in MATLAB (MathWorks, Natick, MA). The first step was to obtain eye velocity, and this was achieved by differentiating the eye position over time and smoothing it with a three-window moving average filter, to reduce the additional noise that arose from the differentiation process.^18^ Following this, the adaptive threshold algorithm described by Behrens et al.^19^ was used to automatically detect saccades during the smooth pursuit. Once the saccades were detected, these were removed from the eye movement traces, and the periods free of saccades were considered smooth pursuit. Further analysis of the smooth pursuit segments followed the procedures described in Vinuela-Navarro et al.^16^ In brief, linear regressions were performed on each segment of smooth pursuit, and the slope of the fitted equation was defined as the eye velocity for that segment. The velocity of each segment was weighted for the duration of the segment, and then velocities were averaged together to obtain the mean time-weighted velocity for that smooth pursuit task and participant. Velocity gain was calculated by dividing the time-weighted mean eye velocity by the stimulus velocity. The total proportion of smooth pursuit was defined as the total amplitude of the eye movement involving slow phase (i.e., without saccades) divided by the total stimulus movement (20° for each smooth pursuit ramp). The number of saccades detected by the algorithm and their amplitude (eye position at the end of saccade subtracted from the eye position at the beginning of the saccade) were also obtained and used to further evaluate the quality of the smooth pursuit. Mean values were obtained from all participants for each age group.

### Statistics

The IBM SPSS software package version 18.0 (IBM SPSS Inc., Chicago, IL) was used for statistical analysis. One-way ANOVA was used to investigate the effect of age on the smooth pursuit performance parameters for each movement condition. Normality tests, including histograms and Shapiro-Wilk tests, were performed on all data. Parametric ANOVA has been suggested to be robust to moderate deviations of normality,^20^ and therefore, this test was used when most of the data (≥50%) were normally distributed and nonparametric ANOVA (Kruskal-Wallis test) was used when most of the data (≥50%) were not normally distributed. For parametric ANOVA, the homogeneity of variances was assessed with Levene's test for equality of variances. A Welch test was used instead of ANOVA for parameters whose variances were not homogeneously distributed. Post hoc tests were performed only when ANOVA or Welch tests were significant. Tukey and Games-Howell post hoc tests were chosen for parameters with their variances homogeneously distributed and those that were not, respectively. A *P* value < 0.05 was considered significant.

Mann-Whitney *U* paired tests with a Bonferroni correction were used to evaluate the differences between age groups when nonparametric ANOVA (Kruskal-Wallis) was significant.

Medians and quartiles (first and third) were calculated for each parameter and age group given that some parameters studied were not normally distributed.

## Results

The eye movement recordings from two children with nystagmus (1 female aged 11 years and 1 male aged 5 years) and two children with strabismus (1 female aged 7 and 1 male aged 5) were discarded from analysis. Similarly, calibration was not successful in two child participants (2 females aged 4 and 7 years), and their eye movements recordings were also discarded from the analysis. In total, the eye movement recordings from 120 children (57 females and 63 males) and 10 adults (5 males and 5 females) were analyzed.

**Figure 1:**
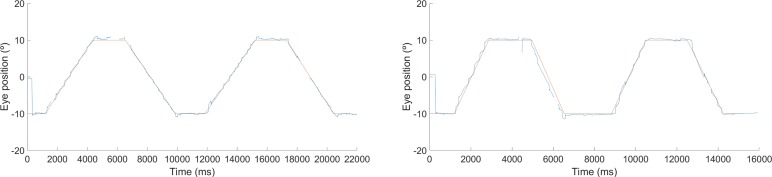
Examples of succesful eye movement traces (blue) obtained from a participant aged 6 years for 6°/s (left) and 12°/s (right) horizontal smooth pursuit . Eye position data were available for all smooth pursuit ramps. The stimulus trace is represented in red.

**Figure 2:**
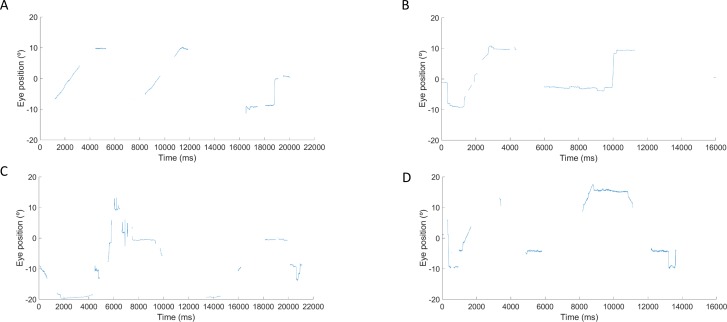
Examples of unsuccesful eye movement traces obtained from different participants aged 6 years for 6°/s horizontal (A), 12°/s horizontal (B), 6°/s vertical (C), and 12°/s vertical (D) smooth pursuit. Eye position data were available from less than two smooth pursuit ramps. These traces were discarded from the analysis.

During data analysis, the researcher discarded trials in which the eye movement trace was poor, so that, if in one of the smooth pursuit presentations a participant produced two or fewer ramps of smooth pursuit, these data sets were discarded from the analysis. Figure 1 shows examples of successful eye movement recordings that were included in the analysis while Figure 2 shows examples of poor eye movement traces that were discarded from the analysis. Successful smooth pursuit data (e.g., >2 smooth pursuit ramps produced) from 119 (99.1%) and 117 (97.5%) children were obtained for the 6°/s and 12°/s horizontal smooth pursuit, respectively. For the vertical condition, successful smooth pursuit data from 115 (95.8%) and 113 (94.1%) children were obtained for 6°/s and 12°/s, respectively. Successful horizontal and vertical smooth pursuit (6°/s and 12°/s) were obtained for all 10 adults (100%). Table 1 provides a summary of the number of child participants in each group and the number of successful recordings for each smooth pursuit condition and age group.

**Table 1:** Summary of the Number of Child Participants in Each Age Group and the Number of Successful Recordings for Each Smooth Pursuit Condition and Age Group

Age, y	*N*	Successful Recordings, 6°/s Horizontal Smooth Pursuit	Successful Recordings, 12°/s Horizontal Smooth Pursuit	Successful Recordings, 6°/s Vertical Smooth Pursuit	Successful Recordings, 12°/s Vertical Smooth Pursuit
4	16	16	16	15	14
5	19	19	19	18	16
6	17	16	16	16	16
7	19	19	19	19	19
8	13	13	13	13	13
9	12	12	11	11	12
10	21	21	20	20	20
11	3	3	3	3	3
Adults	10	10	10	10	10

### Horizontal Smooth Pursuit

Table 2 presents a summary of the horizontal smooth pursuit parameters obtained for each age group.

**Table 2:** Median, Quartiles, Minimum, and Maximum Values for Horizontal Smooth Pursuit Parameters for Children Aged 4 to 11 Years and Adults

Parameter	4 y	5 y	6 y	7 y	8 y
6°/sec horizontal smooth pursuit					
Velocity gain					
Median	0.85	0.87	0.85	0.90	0.88
Quartiles (1, 3)	0.82; 0.88	0.82; 0.89	0.84; 0.89	0.81; 0.92	0.84; 0.91
Min and max	0.65; 0.99	0.65; 0.97	0.73; 0.94	0.76; 0.99	0.79; 0.95
Proportion of smooth pursuit					
Median	0.78	0.77	0.81	0.84	0.82
Quartiles (1, 3)	0.69; 0.83	0.64; 0.83	0.69; 0.83	0.73; 0.88	0.80; 0.88
Min and max	0.52; 0.93	0.50; 0.92	0.55; 0.86	0.64; 0.93	0.60; 0.94
Number of saccades					
Median	6	5	6	6	5
Quartiles (1, 3)	3; 8	3; 12	2; 9	2; 8	3; 7
Min and max	1; 18	0; 18	2; 17	0; 40	1; 9
Amplitude of saccades					
Median	1.49	1.39	2.15	1.38	1.39
Quartiles (1, 3)	1.05; 2.31	1.26; 3.80	1.29; 3.31	1.25; 2.11	1.25; 1.81
Min and max	1; 4.68	0; 11.3	1.12; 19.19	1.03; 7.42	1.06; 9.19
12°/sec horizontal smooth pursuit					
Velocity gain					
Median	0.81	0.83	0.85	0.83	0.85
Quartiles (1, 3)	0.79; 0.84	0.77; 0.89	0.80; 0.90	0.77; 0.90	0.83; 0.90
Min and max	0.63; 1	0.72; 1	0.62; 0.96	0.75; 0.96	0.78; 0.93
Proportion of smooth pursuit					
Median	0.80	0.75	0.83	0.81	0.84
Quartiles (1, 3)	0.71; 0.88	0.72; 0.81	0.77; 0.86	0.76; 0.87	0.78; 0.95
Min and max	0.46; 1	0.65; 1	0.53; 1	0.63; 1	0.61; 1
Number of saccades					
Median	7	11	7	9	8
Quartiles (1, 3)	3; 10	7; 13	5; 10	5.5; 10	7; 10
Min and max	0; 12	3; 15	2; 13	0; 27	2; 10
Amplitude of saccades					
Median	1.92	1.91	2.18	1.80	1.93
Quartiles (1, 3)	1.80; 2.21	1.73; 2.37	1.9; 2.58	1.66; 2.14	1.77; 2.37
Min and max	1.63; 8.83	1.51; 5.36	1.62; 5.37	1.45; 7.6	1.44; 3.58

min, minimum; max, maximum.

**Table 2:** Extended

Parameter	9 y	10 y	11 y	Adults	*P* Value
6°/sec horizontal smooth pursuit					
Velocity gain					
Median	0.89	0.90	0.90	0.93	0.003
Quartiles (1, 3)	0.88; 0.90	0.86; 0.93	0.90; 0.91	0.91; 0.96	
Min and max	0.83; 1	0.82; 0.97	0.88; 0.91	0.88; 1	
Proportion of smooth pursuit					
Median	0.83	0.84	0.82	0.82	0.093
Quartiles (1, 3)	0.80; 0.87	0.80; 0.87	0.80; 0.83	0.80; 0.89	
Min and max	0.78; 0.93	0.58; 0.94	0.75; 0.8.	0.77; 0.96	
Number of saccades					
Median	3	5	3	1	0.128
Quartiles (1, 3)	2; 6	2; 8	3; 4	0; 5	
Min and max	0; 13	0; 22	2; 6	0; 8	
Amplitude of saccades					
Median	1.43	1.3	1.26	1.45	0.450
Quartiles (1, 3)	1.28; 1.70	1.22; 2.77	1.17; 1.76	1.23; 2	
Min and max	1.16; 2.39	1.02; 10.46	1.1; 3.06	1.08; 2.35	
12°/sec horizontal smooth pursuit					
Velocity gain					
Median	0.92	0.86	0.86	0.93	0.031
Quartiles (1, 3)	0.90; 0.94	0.82; 0.91	0.80; 0.96	0.87; 0.95	
Min and max	0.78; 1	0.68; 0.93	0.78; 0.98	0.83; 0.98	
Proportion of smooth pursuit					
Median	0.89	0.81	0.85	0.88	0.366
Quartiles (1, 3)	0.84; 0.94	0.78; 0.87	0.85; 0.87	0.83; 0.94	
Min and max	0.74; 0.98	0.65; 0.91	0.84; 0.91	0.65; 1	
Number of saccades					
Median	6	7.5	7	4.5	0.036
Quartiles (1, 3)	4; 6.5	5; 10	5.5; 7	3; 8	
Min and max	0; 11	0; 12	1; 8	1; 10	
Amplitude of saccades					
Median	1.78	1.76	1.66	1.44	0.016
Quartiles (1, 3)	1.47; 2.63	1.65; 2.06	1.63; 1.69	1.37; 1.68	
Min and max	1.44; 7.73	1.3; 4.34	1.57; 1.73	1.17; 5.13	

#### Velocity gain and proportion of smooth pursuit

**Figure 3:**
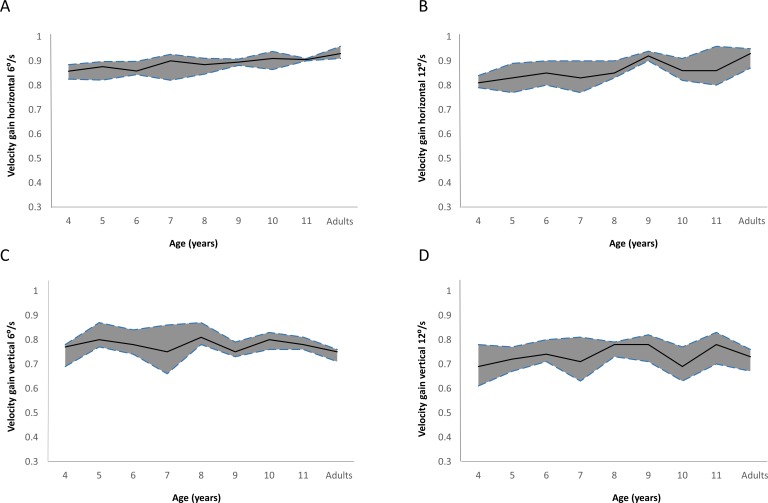
Median velocity gain (solid line) for each age group for 6°/s horizontal (A), 12°/s horizontal (B), 6°/s vertical (C), and 12°/s vertical (D) smooth pursuit. The dashed lines represent the upper and lower quartiles.

As expected, velocity gains for the 12°/s horizontal smooth pursuit were overall significantly lower than for the 6°/s velocity (paired *t*-tests: *t* = 2.710; *P* = 0.008). Figures 3A and 3B illustrate how median velocity gain increases significantly with age until the age of 7 to 8 years (6°/s: *F* = 3.164, *P* = 0.003; 12°/s: *F* = 2.209; *P* = 0.031). Post hoc tests revealed that velocity gain for the 6°/s horizontal smooth pursuit was significantly different between adults and children aged 4 (*P* = 0.010), 5 (*P* = 0.007), and 6 (*P* = 0.029) years. Post hoc tests also revealed significant differences in velocity gain for the 12°/s condition between children aged 5 and 9 years (*P* = 0.042). Table 2 shows that the proportion of smooth pursuit for both the 6°/s and 12°/s horizontal smooth pursuit is close to 0.8 for most age groups, and ANOVA did not show significant differences across ages for this parameter at either velocity (6°/s: *F* = 1.874, *P* = 0.093; 12°/s: *F* = 1.103, *P* = 0.366). In addition, no significant differences were found between the proportion of smooth pursuit for 6°/s and 12°/s (paired *t*-tests: *t* = −1.080; *P* = 0.06).

#### Number and amplitude of saccades

Paired sample *t*-tests revealed that the number of saccades was significantly higher for the 12°/s than for the 6°/s smooth pursuit (*t* = −3.350; *P* < 0.001), but no significant differences between the two velocities were found for the mean amplitude of the saccades (*t* = 0.127; *P* = 0.899). The number and amplitude of saccades during smooth pursuit tended to decrease with age for both horizontal smooth pursuit velocities (Table 2). Interestingly, ANOVA revealed statistically significant differences across ages in the number and amplitude of the saccades for the 12°/s condition (number of saccades: χ^2^ = 16.48; *P* = 0.036; amplitude of saccades: χ^2^ = 18.813, *P* = 0.016), but not for 6°/s (number of saccades: χ^2^ = 12.549, *P* = 0.128; amplitude of saccades: χ^2^ = 7.924; *P* = 0.450). Post hoc tests revealed that, for horizontal 12°/s smooth pursuit, the mean number of saccades in children aged 4 to 7 years was significantly higher than in adults (*P* < 0.001). Similarly, post hoc test also revealed that, for the same smooth pursuit motion, the amplitude of the saccades during the smooth pursuit was significantly larger in children aged 5 and 6 years (*P* < 0.001) compared with the adult group.

### Vertical Smooth Pursuit

Table 3 presents a summary of the vertical smooth pursuit parameters obtained for each age group.

**Table 3:** Median, quartiles, Minimum, And Maximum Values for Vertical Smooth Pursuit Parameters for Children Aged 4 to 11 Years and Adults

Parameter	4 y	5 y	6 y	7 y	8 y
6°/sec vertical smooth pursuit					
Velocity gain					
Median	0.77	0.80	0.78	0.75	0.81
Quartiles (1, 3)	0.69; 0.78	0.77; 0.87	0.74; 0.84	0.66; 0.86	0.78; 0.84
Min and max	0.61; 0.95	0.60; 1	0.70; 0.91	0.56; 0.95	0.71; 1
Proportion of smooth pursuit					
Median	0.55	0.62	0.68	0.64	0.71
Quartiles (1, 3)	0.42; 0.68	0.57; 0.73	0.58; 0.71	0.54; 0.77	0.66; 0.73
Min and max	0.27; 0.91	0.44; 0.86	0.42; 0.91	0.36; 0.96	0.51; 1
Number of saccades					
Median	9	8	8	10	7
Quartiles (1, 3)	6; 19	5; 11	5; 13	5; 17	6; 9
Min and max	2; 60	2; 56	1; 28	2; 28	5; 15
Amplitude of saccades					
Median	2.02	2.19	2.75	1.79	2.18
Quartiles (1, 3)	1.83; 2.75	1.81; 2.93	2.03; 3.76	1.36; 3.70	1.35; 3.27
Min and max	1.23; 6.38	1.32; 10.03	1.16; 6.06	1.16; 6.54	1.16; 6.07
12°/sec vertical smooth pursuit					
Velocity gain					
Median	0.69	0.72	0.74	0.71	0.78
Quartiles (1, 3)	0.61; 0.78	0.67; 0.77	0.71; 0.80	0.63; 0.81	0.73; 0.79
Min and max	0.42; 0.84	0.57; 0.82	0.65; 0.99	0.48; 0.91	0.63; 0.88
Proportion of smooth pursuit					
Median	0.55	0.62	0.68	0.65	0.75
Quartiles (1, 3)	0.38; 0.78	0.48; 0.73	0.66; 0.78	0.53; 0.75	0.71; 0.78
Min and max	0.23; 0.88	0.36; 0.79	0.52; 0.98	0.34; 0.91	0.43; 0.88
Number of saccades					
Median	9	9	7	9	8
Quartiles (1, 3)	5; 12	8; 12	5; 9	6; 13	7; 10
Min and max	0; 26	3; 20	4; 19	1; 17	4; 44
Amplitude of saccades					
Median	2.51	2.02	2.33	2.47	2.36
Quartiles (1, 3)	2.04; 3.31	1.82; 3.89	2.07; 2.99	1.84; 3.14	1.94; 2.76
Min and max	1.48; 17.2	1.66; 6.02	1.74; 9.35	1.36; 6.78	1.67; 12.03

**Table 3:** Extended

Parameter	9 y	10 y	11 y	Adults	*P* Value
6°/sec vertical smooth pursuit					
Velocity gain					
Median	0.75	0.80	0.78	0.75	0.368
Quartiles (1, 3)	0.73; 0.79	0.76; 0.83	0.76; 0.81	0.71; 0.76	
Min and max	0.52; 1	0.62; 0.92	0.75; 0.9	0.68; 0.87	
Proportion of smooth pursuit					
Median	0.63	0.68	0.67	0.58	0.029
Quartiles (1, 3)	0.53; 0.67	0.56; 0.76	0.67;0.69	0.54; 0.62	
Min and max	0.36; 0.93	0.50; 0.81	0.65; 0.71	0.41; 0.71	
Number of saccades					
Median	12	7	13	8	0.492
Quartiles (1, 3)	9; 22	5; 16	9; 22	4; 24	
Min and max	4; 54	2; 48	4; 41	3; 46	
Amplitude of saccades					
Median	1.75	1.63	2.48	1.44	0.450
Quartiles (1, 3)	1.47; 2.30	1.48; 2.28	2.18; 2.64	1.31; 1.66	
Min and max	1.24; 4.08	1.12; 5.34	1.69; 2.76	1.08; 4.14	
12°/sec vertical smooth pursuit					
Velocity gain					
Median	0.78	0.69	0.78	0.73	0.336
Quartiles (1, 3)	0.71; 0.82	0.63; 0.77	0.70; 0.83	0.67; 0.76	
Min and max	0.59; 0.85	0.58; 1	0.60; 0.84	0.61; 0.84	
Proportion of smooth pursuit					
Median	0.73	0.66	0.59	0.70	0.084
Quartiles (1, 3)	0.66; 0.78	0.57; 0.70	0.50; 0.67	0.63; 0.76	
Min and max	0.3; 0.89	0.27; 0.85	0.43; 0.71	0.55; 0.84	
Number of saccades					
Median	8	9	10.5	6	0.593
Quartiles (1, 3)	6; 10	7; 12	8; 18	4.5; 10	
Min and max	0; 25	3; 32	8; 33	4; 15	
Amplitude of saccades					
Median	2.23	2.43	2.44	2.05	0.490
Quartiles (1, 3)	1.89; 2.55	2.12; 4.09	2.15; 2.81	1.65; 2.39	
Min and max	1.69; 4.71	1.78; 10.62	1.69; 3.5	1.34; 3.26	

#### Velocity gains and proportion of smooth pursuit

Similar to the results found for the horizontal smooth pursuit, an increase in stimulus velocity for the vertical smooth pursuit also resulted in significantly lower velocity gains (paired *t*-test: *t* = 4.111; *P* < 0.001), but no significant differences in proportion of smooth pursuit (paired *t*-tests: *t* = 0.269; *P* = 0.788). Figures 3C and 3D illustrate the median velocity gain for vertical smooth pursuit, and in general, it can be observed that median velocity gains lie below 0.8 for both velocities. Moreover, even in adults, this parameter is below 0.8. No trend across ages is evident for the 6°/s and 12°/s vertical velocity gain, suggesting that this parameter does not change with age. These results are confirmed by a nonsignificant ANOVA result for both velocities (6°/s: *F* = 1.101, *P* = 0.368; 12°/s: *F* = 1.150, *P* = 0.336) as shown in Table 3. The proportion of smooth pursuit for the 6°/s and 12°/s conditions tends to increase slightly with age, but in general, the differences across ages are negligible. ANOVA did not reveal any significant differences across ages for the 12°/s vertical condition (*F* = 1.948; *P* = 0.084), but did show significant difference across ages for the 6°/s condition (*F* = 2.431, *P* = 0.029). However, further statistical analysis with post hoc tests revealed no significant differences between any of the child groups and/or adult group.

#### Number and amplitude of saccades

Paired *t*-tests found that the number of saccades is significantly lower for 12°/s than for 6°/s (*t* = −2.332; *P* = 0.022), whereas no differences were found between the two velocities for the amplitude of saccades (*t* = −2.323; *P* = 0.056). Some differences across ages can be observed in the number of saccades for both pursuit velocities (Table 3), but these do not follow a particular trend, and these differences were not significant across ages (6°/s: χ^2^ = 6.417, *P* = 0.492; 12°/s: χ^2^ = 6.487, *P* = 0.593). Similar to the results obtained for the number of saccades, the amplitude of the saccades during the smooth pursuit does not appear to vary across ages (Table 3). ANOVA also confirmed no significant differences in the mean amplitude of saccades across the age groups for 6°/s (χ^2^ = 7.950, *P* = 0.450) and 12°/s (χ^2^ = 7.444; *P* = 0.490).

## Discussion

In general, our findings show that there are some differences in smooth pursuit performance across ages and, therefore, indicate that smooth pursuit is still developing during school age, mainly for the horizontal direction. Indeed, significant differences were found across age groups for horizontal smooth pursuit, showing an increase in smooth pursuit gain and a decrease in saccades during smooth pursuit. These findings suggest an improvement of horizontal smooth pursuit performance with age in school children. In contrast, differences in smooth pursuit across ages on the vertical direction were less evident and most parameters did not show significant differences across ages for this direction. That said, the results obtained in the vertical direction demonstrate that smooth pursuit performance is significantly poorer in this direction than in the horizontal direction. This is found in children of all age groups as well as in adults.

The literature presents conficting results with regard to the smooth pursuit perfomance parameters in children of different ages. Some studies evaluating smooth pursuit in children ranging from 4 years of age to early midadolesence (12 to 15 years) report lower smooth pursuit gains^3^ and higher number of saccades during smooth pursuit^21^ than those found in this study. In contrast, other published results evaluating smooth pursuit in children of specific age groups report very similar smooth pursuit gains to the ones found for the equivalent age groups in our study.^7^,^11^ It has to be noted that direct comparisons of results are difficult as different recording instruments, methods, and procedures are used by different research groups. For instance, most studies investigating smooth pursuit eye movements in children use infrared eye tracking systems (e.g., see Refs. 6, 7) but some have also used video-based tracking systems (e.g., see Refs. 3, 21), photoculography (e.g. see Ref. 11), and electrooculography (e.g., see Ref 5). Discrepancies between results could also be attributed to the fact that this population is strongly affected by the nature of the stimulus.^3^ It has recently been demonstrated that using pictures to elicit smooth pursuit produces higher gains than using dots, particularly for young children.^3^ Suprisingly, the horizontal gains reported in the study using moving but nonanimated cartoons to elicit smooth pursuit are lower than those presented here, but gains similar to ours (of the order of ≥0.8) have previously been found using a continuously changing color dot to elicit smooth pursuit.^11^

There is also some contradiction in relation to the age at which smooth pursuit eye movements reach maturation and adult values. For instance, two studies proposed that this type of eye movement reaches adult values in terms of gains during adolescence.^6^,^7^ However, other studies have found higher gains in children^8^,^11^ suggesting that maturation is complete close to the age of 10 years. Moreover, a study in a large sample of children aged between 6 weeks and 6 years, proposed that smooth pursuit could potentially reach adult values for most parameters by the age of 6 or soon after.^11^ However, the study did not have an adult sample for comparison to investigate this in detail. In order to provide valuable insight into the age at which smooth pursuit reaches maturation, our study included an adult group. Consistent with the suggestion by Rutsche et al.^11^ our results show that, after the age of 7 to 8 years, smooth pursuit parameters are no different from those in adults, and therefore present a strong case to further support an earlier maturation of the smooth pursuit system. To our knowledge, our study and the study by Rutsche et al.^11^ are the only published work in which smooth pursuit eye movements have been investigated in children using a “changing” stimulus, and therefore, it is reasonable to suggest that the use of dynamic and child-friendly stimuli is a critical feature in eliciting improved performance and demonstrating an early smooth pursuit maturation.

Finally, most studies investigating smooth pursuit eye movements in children have recruited their participants through local advertising,^3^,^7^,^21^ but not many have recruited children directly from educational institutions.^5^,^11^ In addition, most studies have focussed on assessing the characteristics of smooth pursuit at particular age groups^5^,^8^,^12^,^21^ rather than assessing these for wider age ranges.^7^,^11^ To our knowledge, this is the first study that presents a detailed characterization of smooth pursuit parameters in typically developing school-age children whose attention has been better engaged by an animated pursuit stimulus. It could therefore be argued that the values presented in this study provide a more appropriate characterisation of the smooth pursuit system in this population because they are derived with a child-friendly setup and will be valuable in future studies that aim to investigate eye movement characteristics in children with suspected eye movement deficits. It is important to note that all data presented here are derived using the Tobii TX300 system. Whether the outcomes are transferable to other recording systems remains to be seen.

### Conclusions

Our findings show that children can produce adultlike, smooth pursuit eye movements as young as 7 years, and strengthen the view that smooth pursuit maturation might be earlier than generally accepted. Our child-friendly approach is a key factor in demonstrating this.
